# Market competition and price of disease management programmes: an observational study

**DOI:** 10.1186/s12913-014-0510-8

**Published:** 2014-10-30

**Authors:** Christel E van Dijk, Bob Venema, Judith D de Jong, Dinny H de Bakker

**Affiliations:** NIVEL, Netherlands Institute for Health Services Research, P.O. Box 1568, 3500 BN Utrecht, The Netherlands; Scientific Centre for Transformation in Care and Welfare (TRANZO), Tilburg University, Tilburg, The Netherlands

**Keywords:** Managed competition, Disease management programmes, Price, Netherlands, Health insurance

## Abstract

**Background:**

Managed competition was introduced into the health care system in several countries including the Netherlands, although effects of competition of both providers and health insurers on the price of health care are inconclusive. We investigated the association between competition of both providers (care groups) and health insurers and the price of disease management programmes (DMPs).

**Methods:**

Data from 76 DMP contractual agreements for type II diabetes mellitus in 2008, 2009 and 2010 were used to analyse the association between market competition and the price of DMPs. Market competition was calculated per municipal health services region (GGD). Insurer market competition was measured by the Herfindahl-Hirschman Index (HHI), care group competition by the number of care groups and the care group market share of GPs. The effect of competition was cross-sectionally studied with linear regression analyses.

**Results:**

Insurer market concentration (HHI) and care group market share were not associated with the price of DMPs. The number of care groups in a GGD region was associated with a lower price (−€4.68; 95% CI: −8.36 - -1.00). The mean difference in the price of DMPs between health insurers was €58.

**Conclusions:**

The price of DMPs seems to be more dependent on the particular health insurer than on market conditions. For competition among health insurers and provider groups to develop, preconditions such as selective contracting and option for patient to change provider should be in place.

## Background

Managed competition in health care has been introduced in several countries [1,2]. Within managed competition health insurers and health care providers are supposed to compete on price and quality of health care within certain rules established by the government to guarantee public objectives. According to neoclassical economics, an increase in competition among providers and health insurers is associated with a decrease in price when selective contracting is an option for health insurers. However, the neoclassical assumptions of perfect information for all actors and certainty do not hold in the health care market [3]. Physicians generally have more information about diseases, diagnostic possibilities and treatment effects than health insurers [4]. Uncertainty exists about the timing of health care and the effects of health care. Also, in neoclassical economics efficient allocation of health care is not necessarily socially desirable, since particularly low income groups will then be excluded from health care [5]. Solidarity and equity are important values in most western countries with public health insurance. These values are important in policy decisions, which counteract the efficient allocation according to the neoclassical theory. For these reasons, effects of managed competition on the price of health care services may differ from the neoclassical response to competition.

Literature on the effects of competition of both (organisations of) providers and health insurers on the price of health care services show inconsistent results [6-12]. Suggested explanations for a lack or opposite effects of competition on prices are the violation of requirements for perfect competition, the relative shortages of health care providers, the lack of eagerness of those insured to switch providers, and the high marketing and advertising costs related to competition [10-12]. These results give policy makers little assistance in developing health policy. More insight into effects of competition on the price of health care services is crucial for evaluating and developing health policy. The introduction of more managed competition in the Dutch health care system provides opportunities to study effects of competition on the prices of services.

In January 2006, the Dutch government introduced The Health Insurance Act based on the principles of managed competition [13]. The new health insurance system gives insurers flexibility to design their products to better appeal to patients and the ability to selectively contract with health care providers as this is thought to improve the efficiency of the health care system [14]. In the new health insurance system, health insurers are obliged to accept all applicants for the same premium, and compete for those insured. The insured can exert pressure on the insurers through their ability to switch health insurance providers [15]. Qualitative information on performance of both insurers and providers and actual different options for the insured are important prerequisites for those insured to make informed choices and exert pressure. Freely negotiable tariffs have been introduced gradually to control for possible undesirable effects, such as price rises or increases in provided services. This gradual introduction is directed by the government. A good example of this gradual introduction is hospital care; the freely negotiable part of costs of hospitals rose from 10% in 2006 to 20% in 2008, 31% in 2009 and to 70% in 2012. Also, part of the tariffs of services of general practitioners (GPs), physiotherapists, primary care psychologists and dentists (from 2012 to 2013) are freely negotiable. Reported effects of managed competition are not always positive. Tariffs for hospital care in the freely negotiable part decreased from 2009 to 2010 by three percent, however this decrease was accompanied with a ten percent increase in volume of services [16]. Experiments with free tariffs for dental care resulted in an increase in tariffs, finally resulting in the preliminary abolishment of the experiment [17,18].

In this article, we investigate the association between competition of both providers and health insurers, and the price of disease management programmes (DMPs) for type II diabetes mellitus. In the Netherlands, DMPs can nationwide be financed by disease oriented funding since 2010 (in Dutch: ‘integrale bekostiging’ – experimental since 2007), also called bundled payments. Within disease oriented funding, care is provided according to the national multidisciplinary evidence-based health care standard agreed between health care providers and patient organisations. Care has to be organised by a group of health care providers (who form a legal entity – hereafter called care groups) who negotiate a lumpsum remuneration per patient with insurers. Care groups are the contracting organisation and not necessarily the health care providers who deliver care. General practitioners (GPs) play a central role in care groups [19-21]. Care groups can either deliver (part of) the care themselves or subcontract with other health care providers (e.g. dieticians, medical specialists). In the Netherlands, every discipline of health care providers in curative health care has their own payment system. However, disease oriented funding is a payment system of curative health care across different disciplines. Without a contract for DMPs between care groups and insurers, care is financed per discipline.

This study aims to determine the association between competition among care groups and health insurers, and the price of DMPs, by conducting a cross-sectional study of 76 DMP contractual agreements for type II diabetes mellitus. We expected the price of DMPs for diabetes mellitus patients to be lower with increased competition between care groups and health insurers, with the lowest price in regions with both markets regarded as competitive.

## Methods

### Study design

This was a cross-sectional, observational study analysing the association between competition among care groups and health insurers, and the price of DMPs for type II diabetes mellitus in the Netherlands. Data were used from 76 contractual agreements^a^ for the Dutch type II diabetes mellitus DMP for 2008 (N = 8), 2009 (N = 33) and 2010 (N = 35). Some contracts with care groups are signed for more than one year with 2008 or 2009 as the most recent contract available. One hundred nine care groups operate in the Netherlands, making this a sample of 70% of all possible contractual agreements [22]. Competition between care groups and health insurers was assessed at the level of municipal health services regions (GGD-regions). GGD regions were selected as these regions are expected to provide a regional overview of competition among care groups, as well as, the ability to calculate the insurer concentration in these regions. There are 31 GGD regions in the Netherlands, these regions are defined by postal codes, with on average 530,000 inhabitants (min: 164,000; max: 1,000,000). According to national data protection guidelines ethical approval by an ethics committee is unnecessary.

### Data collection

Information on the price of DMPs was requested from health insurers. The care purchasing department of eight insurance companies were approached by telephone with the request to provide contract data for this study. A document containing the aim and methods of the study was e-mailed to insurers who requested additional information. Two out of eight insurers agreed to participate in the study. One insurer sent all their contracts via e-mail; the contracts of the other insurer were viewed at the insurance company. Although, only two out of eight insurers participated, we were able to include contracts of seven health insurers. In the Netherlands, the tendency of health care providers was to negotiate with the health insurer with the highest market share and to propose this contract to the other health insurers in the region. In the time period 2008–2010, a lot of health insurers agreed upon the proposed contract of another health insurer without additional precondition or changes in tariffs. So, the two health insurers participating in our study agreed with contracts and prices of DMPs made by other health insurers. For this reason, contracts of other health insurers were included also. The two insurers supplied data of 76 contracts from seven insurers.

Data regarding the characteristics of the 109 Dutch care groups in 2010 was obtained from previous studies [22]. The care group data contains among others the care groups’ city of residence and the number of GPs per care group (available for 63 groups). 2009 data regarding the patient insurer in the GGD regions (to estimate insurer market concentration) was obtained from the NIVEL ‘Dutch Health Care Consumer Panel’ (N = 994) [23]. The Dutch ‘General Practitioner Registration’ (in Dutch: huisartsenregistratie) was used to gather the number of GPs per GGD region in 2009 [24].

### Measures

#### Dependent variable

The dependent variable in the analyses was the price per diabetes patient in Euros for a DMP as formulated in the contractual agreements between health insurers and care groups. The price of DMP was normally distributed (Skewness-Kurtosis test p = 0.5471).

#### Independent variable

Three variables were used to quantify the market structure within the GGD regions: insurance market concentration, the number of care groups, and the care group market share.

1. Insurer market concentration was measured with the Herfindahl-Hirschman Index (HHI) and based on data from the Dutch Health Care Consumer Panel. The index consists of the sum of the squared insurer market shares for all insurers in the GGD regions and ranges from 0 to 10,000. A higher Herfindahl signifies a less competitive market. Insurance holdings’ daughter companies were recoded into the title of the negotiating insurer. The HHI was calculated for 30 of 31 Dutch GGD regions. For one GGD-region the HHI-index could not be calculated, as no consumer panel data were available for this region. These cases were excluded.
2. The number of care groups in a GGD region was based on data of Dutch care groups.
3. The care group market share of GPs was calculated by dividing the number of GPs per care group by the total number of GPs in the GGD region in which the care group is active, and ranges from 0–1. No HHI was calculated since no information was available about the number of diabetes mellitus type II patients per region and per care group.

### Statistical analyses

To analyse the association between competition of care groups and health insurers and the price of DMPs, linear regression analyses were conducted. The dependent variable in the analyses was the price of DMPs. The independent variables were insurance market concentration, the number of care groups and care group market share. Univariate and multivariate linear regression analyses were performed. In the multivariate linear regression analyses all independent variables were forced in the model, regardless of the significance level. Separate multivariate linear regression analyses were performed for both measures of care group competition. Analyses were adjusted for the percentage of persons with a low income (lowest 40% of the whole country) and the percentage of persons with a western and non-western nationality in a GGD region. To test whether the specific health insurer influenced the association between market competition and prices, univariate linear regression analyses were also separately performed for insurers with twelve or more contracts (four insurers). The effect of health insurer could not be estimated with a multilevel/mixed model, since the insurers were partly regionally divided. All analyses were conducted using the statistical software STATA (version 10.0). Significance level was set at p < 0.05.

## Results

The average price of a DMP was €353.4 with a standard deviation of 50.0. The median insurer market concentration was 2717 (IQR: 2302–3219), an oligopsony (a market with only a few large buyers, here health insurers). The number of GPs in a care group varied widely, with a median of 50 (IQR: 25–107) GPs. Care groups had a median market share of 16% (IQR: 8-39%) in their region.

Table 1 presents the univariate and multivariate linear regression analyses of the association between competition of care groups and health insurers and the price of DMPs. Only a higher number of care groups in a region was associated with a lower price of DMPs in the univariate regression analyses. In the multivariate linear regression analysis this association was also found. The effect size of -€4.68 per extra care group in the region (average of five care groups; min: 1, max: 13) is limited.

**Table 1:** **Association between competition of care groups and health insurers, and price of DMPs based on linear regression analyses** ^**#**^

	**Univariate linear regression**	**Multivariate linear regression** ^**$**^	
	**Effect (95% CI)**	**Effect (95% CI)**	**Effect (95% CI)** ^**§**^
Insurer market concentration (HHI)	0.004 (−0.008-0.017)	0.009 (−0.005-0.024)	0.009 (−0.005 – 0.024)
Number of care groups in region	−4.78 (−8.41 - -1.15)*	−4.68 (−8.36 - -1.00)*	
Care group market share^$^	0.49 (−0.16 – 1.15)		0.37 (−0.31 – 1.05)

^#^Analyses were adjusted for the percentage of persons with a low income (lowest 40% of the whole country) and the percentage of persons with a western and non-western nationality in a GGD region; ^$^based on 63 care groups since no data were available on the number of GPs for 13 care groups; ^§^Total variance explained (R^2^): 9% and 10%; *p < 0.05.

To explore the relationship between insurer market concentration and care group market share, we divided contractual agreements into four groups depending on the concentration of insurer market and care group market share (Table 2). The four groups were based on an equal distribution and not with respect to content, since otherwise the power in some groups would have been too limited.

**Table 2:** **Distribution of contractual agreements on insurer market concentration and provider market share** ^**$**^

	**HHI insurer market >2700**	**HHI insurer market ≤2700**
Care group market share >16%	Both markets concentrated^a^ (n = 18)	Care group dominant market^c^ (n = 13)
Care group market share ≤16%	Insurer dominant market^b^ (n = 18)	Both markets competitive^d^ (n = 14)

^$^Based on 63 care groups since no data was available on the number of GPs for 13 care groups; ^a^‘Both market concentrated’ refers to the situation where both the care group market and the insurer market is less competitive; ^b^‘Insurer dominant market’ refers to the situation where the insurer market is less competitive, but the care group market is more competitive; ^c^‘Care group dominant market’ refers to the situation where the insurer market is more competitive, but the care group market is less competitive; ^d^‘Both market competitive’ refers to the situation where both the insurer market and the care group market are more competitive.

Table 3 shows the results of the linear regression analyses with as reference group contractual agreements in a region with both a concentrated insurer and care group market. No significant difference in the price of DMPs was found between the four competition groups.

**Table 3:** **Association between the four competition groups and price of DMPs based on multivariate linear regression analysis** ^**$,#,§**^

	**Linear regression**
	**Effect (95% CI)**
Both markets competitive (reference)	
Care group dominant market	−1.70 (−42.50 – 39.11)
Insurer dominant market	−9.19 (−46.88 – 28.50)
Both markets concentrated	26.32 (−10.88 – 63.52)

^#^Analyses were adjusted for percentage of the persons with a low income (lowest 40% of the whole country) and the percentage of persons with a western and non-western nationality in a GGD region; ^$^based on 63 care groups since no data was available on the number of GPs for 13 care groups; ^§^Total variance explained (R^2^): 10%.

Table 4 presents the univariate linear regression analyses of the association between competition of care groups and health insurers and the price of DMPs per health insurer (only for health insurers with twelve or more contracts). These results substantially differ from the results where all health insurers were included (see last column in Table 4), indicating that the associations were largely influenced by health insurers. For all health insurers together, the number of care groups in region affected the price of DMPs negatively, whereas opposite associations were found for three out of four health insurers, although non-significant. It seems that prices are set by health insurers instead of influenced by market conditions. Analyses on the effect of health insurers on the price of DMPs confirm this indication, with a mean difference in price between health insurers of €58.

**Table 4:** **Association between competition of care groups and health insurers, and price of DMPs per health insurer based on univariate linear regression analysis**

	**Health insurer 1 (n = 12)**	**Health insurer 2 (n = 25)**	**Health insurer 3 (n = 14)**	**Health insurer 4 (n = 14)**	**All health insurers**
	**Effect (95% CI)**	**Effect (95% CI)**	**Effect (95% CI)**	**Effect (95% CI)**	**Effect (95% CI)**
Insurer market concentration (HHI)	−0.005 (−0.033-0.024)	−0.001 (−0.012-0.009)	−0.002 (−0.015-0.010)	−0.003 (−0.013-0.007)	0.004 (−0.008-0.017)
Number of care groups in region	5.66 (−17.29-28.62)	2.04 (−1.81 – 5.90)	2.45 (−0.73-5.63)	−0.51 (−2.30-1.28)	−4.78 (−8.41 - - 1.15)*
Care group market share	0.15 (−2.49 – 2.80)	0.12 (−0.29-0.53)	0.23 (−0.34-0.79)	0.18 (−0.46-0.82)	0.49 (−0.16 – 1.15)

*p < 0.05.

## Discussion

This study examined the association between health insurer and care group market competition and the price of DMPs. The insurer market concentration was not associated with the price of DMPs, contrary to the expectation based on neoclassical economics. Care group competition, measured as the number of care groups in a GGD region, was associated with a lower price: −€4.68 with an additional care group in the region. As prices differed more strongly between health insurers, the price of DMPs seems to be more dependent on the particular health insurer than on the market conditions.

Results of our study are not necessary applicable to other countries, as they are highly dependent on the health care system. Possible explanations for our findings could be found in limited selective contracting of health insurers, negotiations of care groups with the largest health insurer in the region, the lack of experience with competition among care groups and health insurers, and the relative absence of options for patients to change care group. Health insurers are allowed to selectively contract health care providers [25]. Despite this opportunity, health insurers did not selectively contract health care providers until the autumn of 2010 [26]. So, health insurers seem not to have used their important instrument of selective contracting in the negotiation process with care groups. This may explain the relatively small association between care group competition and the price of DMPs. Since 2010, increasingly health insurers have started selective contracting with health care providers, which in the future may result in a greater association between care group competition and the price of DMPs [26]. On the other hand, care groups with a high market share also hardly used their market power to attain a higher price for their DMP. It could be that health insurers offered a good price for the DMPs of care groups and care groups did not feel the need to negotiate a higher price. In addition, a recent Dutch report showed that health insurers work with calculation models to make financial agreements [27]. Not in all calculation models did care include of all involved health care providers, such as dieticians. This may explain the large difference in the price of DMPs between health insurers.

Care groups often negotiate with the insurer with the highest number of insured in their region. In this way, benefits from negotiations from one health insurer are transferred to other health insurers, which may not give health insurers incentives to negotiate lower prices in a region in which they have more market power, unless substantial gains could be obtained. Increasingly, health insurers start rejecting contractual agreements for DMPs from other health insurers [28]. This might result in more variation in prices between regions with different levels of care group and insurer market competition. However, it might not be realistic to expect extremely different contracts for one care group, since the number of care groups in a region is still limited and so is the room to selectively contract DMPs for health insurers.

Both care groups and health insurers may have lacked experience in the negotiation process of DMPs and therefore prices may differ greatly between health insurers and less so with market competition. Disease oriented funding was experimentally introduced in 2007 and nationwide in 2010. The organisation and management of care groups is still in its infancy. Care groups rarely select health care providers based on their performance and poor performing providers are rarely excluded from care groups [29]. Including poor performers may result in reduced process and patient outcomes and may impede a good negotiation position for care groups. Previous research showed that effects of provider competition need time to develop [6-8]. Effects of care group and health insurer competition may impact more on the price of DMPs in a couple of years and prices may converge between health insurers.

An important aspect in health care systems with managed competition is patient choice. Those insured should be able to make informed choices in selecting their health insurance policy and health care providers. Currently, patients do not always know whether they take part in a DMP and options to change a care group are often restricted since the number of care groups in a region is often limited [29,30]. Therefore, patients can hardly exert pressure on insurers by switching health insurance provider. This may result in less eagerness by insurers to selectively contract or to put pressure on care groups. In addition, selective contracting of care groups may force patients to change GPs, since GPs currently form the basis of care groups. Patients may be more loyal to their GP than insurer, and therefore insurers could lose patients when selective contracting, making them less eager to selectively contract.

### Limitations of this study

This study has some drawbacks. First, contracts may have differed with respect to included health care services. Although care is provided according to the national multidisciplinary evidence-based health care standard, some care groups and health insurers could have agreed upon additional services. Unfortunately, we did not have full information on the exact content of the contracts, which could have influenced effects in both directions. Second, this study does not take regional differences in diabetes severity and its influence on the contract price into account. For instance, a region with a high proportion of diabetes patients on insulin treatment is expected to result in a higher contract price than a region with a low proportion of these patients. This could have influenced our results in both directions. Last, the working area of a care group has been grouped into GGD regions, based on the statutory place of registration. This way of grouping does not take into account the exact field of activity of the care groups. Competition arises when a patient has the possibility to change health care provider within an acceptable distance, which might be a smaller distance than within the GGD regions. Our approach results in a regional competitive level which does not have to correspond with the level of competition between care groups in reality. Previous studies show that the definition of markets can influence effects in both directions [31].

## Conclusions

The present study showed few economic benefits of care group and health insurer competition on the price of DMPs. Health insurer competition was not associated with price of DMP and care group competition was associated with a limited lower price of DMPs. The price of DMPs seems to be more dependent on the particular health insurer than on the market conditions.

## Endnote

^a^The most recent available contract was selected per care group.
